# Supplementary material for: Is examining children and adolescents with autism spectrum disorders a challenge?—Measurement of Stress Appraisal (SAM) in German dentists with key expertise in paediatric dentistry
Source: PLoS One. 2022 Aug 3;17(8):e0271406. doi: 10.1371/journal.pone.0271406 (PMC9348685; doi:10.1371/journal.pone.0271406)
Supplement: Attachment — Submitted filename: Response to Reviewers.docx [file pone.0271406.s001.docx]

Dear Professor Kielbassa, dear reviewers,

we would like to thank you for the helpful notes and comments on our submitted manuscript. We hope that we were able to meet the editorial requirements of the journal as well as the needs of the reviewers to improve the manuscript in our revision.

**Journal Requirements:**

When submitting your revision, we need you to address these additional requirements.

**1. Please** ensure that your manuscript meets PLOS ONE's style requirements, including those for file naming. The PLOS ONE style templates can be found at

https://journals.plos.org/plosone/s/file?id=wjVg/PLOSOne_formatting_sample_main_body.pdf and

https://journals.plos.org/plosone/s/file?id=ba62/PLOSOne_formatting_sample_title_authors_affiliations.pdf

**2. Please** amend your current ethics statement to address the following concerns:

a) Did participants provide their written or verbal informed consent to participate in this study?

b) If consent was verbal, please explain i) why written consent was not obtained, ii) how you documented participant consent, and iii) whether the ethics committees/IRB approved this consent procedure.

- **We amended the current ethics statement (Ethics approval and written consent to participate before the online survey or for publication) in the manuscript.**

**3. Thank** you for stating the following financial disclosure:

“The authors declare that the study was funded by the Department of Special Care Dentistry at Witten/Herdecke University and the Department of Child and Adolescent Psychiatry, Psychotherapy and Neurology of Childhood and Adolescence at the Gemeinschaftskrankenhaus

Herdecke as part of a collaborative project between the two departments. This scientific project is financially supported by the Software-AG-Foundation based in Darmstadt/Hesse, Germany.”

Please state what role the funders took in the study. If the funders had no role, please state: "The funders had no role in study design, data collection and analysis, decision to publish, or preparation of the manuscript." If this statement is not correct you must amend it as needed.

Please include this amended Role of Funder statement in your cover letter; we will change the online submission form on your behalf.

- **We stated “The funders had no role in study design, data collection and analysis, decision to publish, or preparation of the manuscript”. We included this amended Role of Funder statement in the cover letter.**

**4. In your** Data Availability statement, you have not specified where the minimal data set underlying the results described in your manuscript can be found. PLOS defines a study's minimal data set as the underlying data used to reach the conclusions drawn in the manuscript and any additional data required to replicate the reported study findings in their entirety. All PLOS journals require that the minimal data set be made fully available. For more information about our data policy, please see http://journals.plos.org/plosone/s/data-availability.

Upon re-submitting your revised manuscript, please upload your study’s minimal underlying data set as either Supporting Information files or to a stable, public repository and include the relevant URLs, DOIs, or accession numbers within your revised cover letter. For a list of acceptable repositories, please see http://journals.plos.org/plosone/s/data-availability#loc-recommended-repositories. Any potentially identifying patient information must be fully anonymized.

Important: If there are ethical or legal restrictions to sharing your data publicly, please explain these restrictions in detail. Please see our guidelines for more information on what we consider unacceptable restrictions to publicly sharing data: http://journals.plos.org/plosone/s/data-availability#loc-unacceptable-data-access-restrictions. Note that it is not acceptable for the authors to be the sole named individuals responsible for ensuring data access.

We will update your Data Availability statement to reflect the information you provide in your cover letter.

- **Due to the strict European General Data Protection Regulation and the statement in the questionnaire to the participants that no pseudonymised data will be passed on to third parties, we would like to consult with our cooperation partner about the form in which the data could be delivered. An exchange with our cooperation partner on how the data is representated and an adequate creation of a data set based on this could not be completed so far, among other things due to the time available. From the point of view of the involved cooperation partners, this can only be definitely completed when an acceptance of the manuscript has been confirmed.** **We, the authors, thank you, the journal, editor and reviewers, for your understanding in this regard.**

**5. PLOS** requires an ORCID iD for the corresponding author in Editorial Manager on papers submitted after December 6th, 2016. Please ensure that you have an ORCID iD and that it is validated in Editorial Manager. To do this, go to ‘Update my Information’ (in the upper left-hand corner of the main menu), and click on the Fetch/Validate link next to the ORCID field. This will take you to the ORCID site and allow you to create a new iD or authenticate a pre-existing iD in Editorial Manager. Please see the following video for instructions on linking an ORCID iD to your Editorial Manager account: https://www.youtube.com/watch?v=_xcclfuvtxQ.

- **The corresponding author, Daniela Reis, validated her ORCID iD in Editorial Manager.**

**Reviewers' comments:**

Reviewer's Responses to Questions

Comments to the Author

**1. Is the manuscript technically sound, and do the data support the conclusions?**

The manuscript must describe a technically sound piece of scientific research with data that supports the conclusions. Experiments must have been conducted rigorously, with appropriate controls, replication, and sample sizes. The conclusions must be drawn appropriately based on the data presented.

Reviewer #1: No	Reviewer #2: No	Reviewer #3: Yes

- **We have refined the conclusions in the revision and aligned them with the objectives of the study.**
- **Taking into account the result that the treatment of children and adolescents with autism spectrum disorder is seen as a challenge, we pointed out the need to improve dental study in Germany in the field of special needs dentistry.**
- **In addition, we address the usefulness and validity of the SAM in our sample with dentists, who often examinate children and adolescents.**

**2. Has the statistical analysis been performed appropriately and rigorously?**

Reviewer #1: Yes	Reviewer #2: I Don't Know	Reviewer #3: I Don't Know

**3. Have the authors made all data underlying the findings in their manuscript fully available?**

The PLOS Data policy requires authors to make all data underlying the findings described in their manuscript fully available without restriction, with rare exception (please refer to the Data Availability Statement in the manuscript PDF file). The data should be provided as part of the manuscript or its supporting information, or deposited to a public repository. For example, in addition to summary statistics, the data points behind means, medians and variance measures should be available. If there are restrictions on publicly sharing data—e.g. participant privacy or use of data from a third party—those must be specified.

Reviewer #1: No	Reviewer #2: No	Reviewer #3: Yes

- **Due to the strict European General Data Protection Regulation and the statement in the questionnaire to the participants that no pseudonymised data will be passed on to third parties, we would like to consult with our cooperation partner about the form in which the data could be delivered. An exchange with our cooperation partner on how the data is representated and an adequate creation of a data set based on this could not be completed so far, among other things due to the time available. From the point of view of the involved cooperation partners, this can only be definitely completed when an acceptance of the manuscript has been confirmed.** **We, the authors, thank you, the journal, editor and reviewers, for your understanding in this regard.**

**4. Is the manuscript presented in an intelligible fashion and written in standard English?**

PLOS ONE does not copyedit accepted manuscripts, so the language in submitted articles must be clear, correct, and unambiguous. Any typographical or grammatical errors should be corrected at revision, so please note any specific errors here.

Reviewer #1: Yes	Reviewer #2: No	Reviewer #3: Yes

- **We had the manuscript proofread by a native speaker,** **among other things.**

**5. Review Comments to the Author**

Please use the space provided to explain your answers to the questions above. You may also include additional comments for the author, including concerns about dual publication, research ethics, or publication ethics. (Please upload your review as an attachment if it exceeds 20,000 characters)

**Reviewer #1: Abstract**

**Thank you very much for your review. In the following we respond to your comments. We have accepted nearly all comments and implemented the changes in the manuscript.**

- "(...) sent to members of the German Society of Paediatric Dentistry (DGKiZ) via a link to participate." Please add number of members having been invited to participate.

- **In the abstract we add the number of members having been invited to participate.**

- "92 participants (11 male, 81 female) fully completed the questionnaire." Must read "Ninety-two participants (...)", please re-edit. Indeed, 92 would seem a poor number only. Why do you think that this would result in some kind of representative outcome?

- **We re-edit “Ninety-two participants…**
- **We only recruited the participants online. For further projects we think it would make sense to recruit in two ways online and e.g. at conferences. At the beginning of the data cleansing, we had over 190 participants. Due to a high number of missing values, many participants were not included in the analysis. Due to the response rate, the results of the SAM are not representative of all German dentists, but it offers an insight into topics in special needs dentistry in Germany that have not yet been examined.**

- "(...) and in some cases there were high intercorrelations between the scales, (...)." Please provide exact results, give r and p values. Phrases like "some cases" or "high intercorrelations" would not seem acceptable. Please remember that future readers will decide switching to your full text AFTER having read your Abstract section.

- **We have given specific values for the intercorrelations in the abstract.**

- "This could be reasoned in the circumstance that the participants in the present study were generally very experienced in treating children, including children e.g. with underlying diseases." This would not seem astonishing. Do you see any news from your study, not only confirmative ones?

- "In future, dentists who rarely treat children could also be surveyed in a further, larger study." Do not stick to meaningless phrases here. With your Conclusions, please stick exclusively to your revised aims (see comments given below). Do not simply repeat your results (or even speculate) here. Instead, provide a reasonable and generalizable extension of your outcome.

- **In the abstract, we have focused on results, discussion and conclusion with regard to the previous aims.**

**Intro**

- This study has been finished, right? "The purpose of the present study is to check (...)" must read "The purpose of the present study was to check (...)".

- **We re-edit “The purpose of the present study was to check.(…)”.**

- Same with "(...) is examined."

- **We also re-edit- (…) was examined.”**

- Both aims and objectives elaborated here would seem convincing. However, "SAM’s utility" aspects should be mentioned with your Abstract section, too. Please revise/adapt carefully.

- **We also mentioned the “SAM’s utility” aspects in the abstract.**

**Meths**

- Do not use legal terms like GmbH, ®, and so on.

- **We re-edited legal term of the statistic software.**

- With ALL materials and methodologies (including statistical software), please use general names with your text, followed by (brand name; manufacturer, city, ST [if US], country) in parentheses. Stick to semicolon. Revise thoroughly.

- **We revised the text passages thoroughly.**

- "Before the start of the study, a positive vote for carrying out the survey was obtained from the board of the DGKiZ." What about the positive vote of an Ethical Committee? Please provide vote number and date of approval.

- "Since the data collection was planned in accordance with the European General Data Protection Regulation and represents an expert survey, a formal ethics application to the responsible ethics committee of the Witten/Herdecke University was not performed before the start of the project." This would seem astonishing. An "expert survey" does not need an ethical approval? Please provide a sound and reliable reference.

- **We have discussed this with the ethics committee of the Witten/Herdecke University and have received written confirmation that there was no need for professional advice and for ethical approval in the case of anonymous surveys of employees in the healthcare sector. As requested, we have included a previous and similar study as a reference in the manuscript.**

(Reis et al. Erfassung und Verarbeitung von Stress bei ärztlichem Personal in Krankenhäusern der Grund- und Schwerpunktversorgung in Deutschland im zeitlichen Verlauf der COVID-19-Pandemie. Nervenheilkunde. 2022; 41:27–35. doi: 10.1055/a-1650-2173.)

- "(...) successfully sent to 1.725 e-mail addresses (...)." Please see comments given above. With 92 respondents, some 5% have participated only, and this would not seem a reliable database, don't you think so?

- **See the comments under abstract. We also addressed these comments briefly in the discussion section.**

- "(...) This measures cognitive processing mechanisms and perceived stressfulness in the event of acute stress [26]. Peacock and Wong see the transactional stress model as the basis of their questionnaire [34,35]. According to this model, stress arises from (...)." This does not seem to refer to your methodology, but might be discussed later. Please revise carefully, and focus on the methodology here.

- **We have placed this in the methods section because the model, the scales by Peacock and Wong, their names and their meaning must be clarified in advance so that the results can be understood.**

- Reduce repeated mentioning of Author names, please (see, for example, "Peacock and Wong", but this also refers to other names). Instead, please focus on your main thoughts, to ensure readability (Authos' previous work will be acknowledged with your References section).

- **We reduced mentioning of Author names.**

- Legend Fig 1: Please do not repeat the full reference here, see comments given above. [Number] will be sufficient.

- **We only considered the reference numbers in the legend of the figure.**

- Same with legend of Table 2 and Table 4.

- **We only considered the reference numbers in the legends of Table 2,3 and Table 4.**

**Results**

- Revise carefully for any typos. See, for example, Table 1, "(e.g. students. persioner)".

- **We revised typos.**

- "The Kaiser-Meyer-Olkin coefficient as a measure of sample suitability was .80. Since this value was above the lower limit of .50, a factor analysis could be carried out [37]." Please provide more information on the "suitability". Again, see comments given above, and remember that 92 respondents would not seem convincing.

- **We have to qualify that the reliability of a factor analysis also depends on the sample size.**

- Please double check, and revise for correct inter punctuation. See "(β = .64. R2 = .41 p <.01)".

- **We revised for correct inter punctuation.**

- Again, several aspects should be explained and discussed in the Discussion section. Here, please focus on the results.

- **We have included text parts of the results chapter in the discussion**

**Disc**

- "To our best knowledge, there is no study that specifically deals with the psychological and physical stress on dentists from Germany when treating children and adolescents with ASD." It would seem unclear why "dentistry from Germany" have been considered important for the Authors. What would predispose "German dentists" from other nations? What about the transferability of your outcome to other dentists from other countries?

- **We removed the sentence “To our best knowledge, there is no study that specifically deals with the psychological and physical stress on dentists from Germany when treating children and adolescents with ASD” from the manuscript.**

- "This study now offers this information for the first time." And now? Again, why do you think that his would be important?

- **We removed the sentence from the manuscript.**

- Again, please revise for minor shortcomings, see "Since the majority of our study participants already had of professional experience of (...)."

- **We re-edit the typo.**

**Concl**

- Remember that this section is not a second summary. Again, with your Conclusions, please stick exclusively to your revised aims. Do not simply repeat your results (or even speculate) here. Instead, provide a reasonable and generalizable extension of your outcome, which must be based on your results.

- The current version of this section would seem right, but major aspects must be provided with the Discussion section, since these are not considered conclusions from your study.

- **We reconciled the content of the discussion part and the conclusions and wrote parts of the conclusions in the discussion.** **The conclusions are now more detailed and based more strongly on the results.**

**Refs**

- Please stick to the Journal guidelines, and consults some recently published Plos One papers.

- Style would be "Tanagawa M, Yoshida K, Matsumoto S, Yamada T, Atsuta M. Inhibitory effect of antibacterial resin composite against Streptococcus mutans. Caries Res. 1999; 33(5): 366–371. https://doi.org/10.1159/ 000016535 Titel anhand dieser DOI in Citavi-Projekt übernehmen PMID: 10460960 Titel anhand dieser Pubmed-ID in Citavi-Projekt übernehmen" Revise thoroughly.

- **We adjusted the citation style.**

In total, this submitted draft would seem interesting, is considered easily intelligible, and should be worth following after a thorough revision, following the aspects given above. Additionally, the manuscript is ready for external review.

**Reviewer #2:** Many thanks for asking me to review this paper.

**Thank you very much for your review. In the following we respond to your very helpful comments.**

**Overall summary**

While I acknowledge there is little data available on this topic in Germany, overall I do not feel this paper merits publication in a prestigious journal like PLOS ONE. The questionnaire response rate of 5% is inadequate and liable to survey error and a high degree of reporting bias.

- **We considered in the abstract and in the discussion: “Due to the response rate, the results of the SAM are not representative of all German dentists, but it offers an insight into topics in special needs dentistry in Germany that have not yet been examined**

It may have been more prudent for the investigators to find new ways of engaging with the survey population to yield an improved response rate to the survey, rather than attempt to publish a questionnaire survey with such a poor response rate. As almost 95% of the survey population did not respond, I do not feel any credible conclusions can be drawn on the views of German paediatric dentists towards treating children and adolescents with autism spectrum disorders.

- **We considered this in the discussion: We only recruited the participants online. For further projects we think it would make sense to recruit in two ways online and e.g. at conferences. At the beginning of the data cleansing, we had over 190 participants. Due to a high number of missing values, many participants were not included in the analysis. Due to the response rate, the survey is not representative of German dentists, but it offers an insight into topics in special needsdentistry in Germany that have not yet been examined.**

I have some further more specific comments to share below that may help to improve the paper, should attempts be made to improve its methodological rigour:

The title of the paper could be revised to represent the fact that it is a questionnaire study and restricted to paediatric dentists.

- **We revised the title of the paper and e.g. added the restriction to the surveyed profession of German dentists. The specification “(SAM)” already describes the methodology (questionnaire), since the SAM is known as a corresponding tool in e.g. psychology, psychiatry and therefore does not need to be named more specifically as questionnaire study.**

The paper is difficult to follow and would benefit from a technical restructure to provide a clearer rationale for the research question, clearer explanation of background context and greater clarity over terms used to define the population of interest and why this particular methodology was chosen. There may be more appropriate methodologies for investigating this topic and it doesn’t feel like this approach has elucidated the views of German paediatric dentists in a complete and comprehensive way. The methodology appears overly complex for such predictable results, which leads me to think why this research was necessary.

- **The methodology has been chosen deliberately, as there has been no study to date that has investigated the question of whether the examination of persons with ASD is challenging or, if necessary, should be viewed differently (e.g., anxiety-provoking, threatening). Based on your advice, we have revised and structured the manuscript with regard to the research questions, the aims and the applied analyses.**

I fully appreciate the authors are using a second language, but in places it is not clear what is meant, in particular in the background are references being made specifically in regard to autism spectrum disorders or disability/mental disorders more generally?

- **We corrected the references notices related to information about Autism Spectrum Disorders and disabilities/mental disorders in the background section.**

In terms of the methods there are insufficient details of the questionnaire development (piloting and refinement of the tool), administration (for example how were duplicate entries managed, or ineligible individuals accounted for), anonymity, storage and management of the data, and there is no reference to efforts to maximise the response rate. A recruitment diagram would aid reader understanding of the process and response and completion rates. Particularly as the response rate is only referred to in the conclusion section. In addition, a copy of the questionnaire should be provided as an online appendix to accompany the paper.

- **We considered in the method part: In order to be able to compare the results, the development of the questionnaire was based on previous national and international studies”.**
- **We also considered administration matters.**
- **We have already referred to the response rate in the correction in the abstract and in the discussion section.**
- **In this manuscript we have only addressed a small part of the many questions in the questionnaire. Our focus was on the question of the challenge or threat of treating patients with Autism Spectrum Disorders. For this we used the SAM-questionnaire. A few demographic data were also addressed in the manuscript. In our opinion, including the entire questionnaire as an online appendix in the manuscript would tend to overload the reader with additional unnecessary information, which are not adressed in the manuscript.**

The rigour of the results and corresponding conclusions can only be appraised based on a more robust response rate, which is not observed in this work.

- **Please see the comments above.**

**Reviewer #3: Summary**

The manuscript is well written and particularly scientifically sound as the results of the study are given in elaborate detail. The data analysis is very extensive and the regression analysis is discussed in great depth. The study clearly highlights the novel element of using the SAM as a survey tool for measuring stress perceptions and stress appraisal.

**Thank you for your review. We are glad to hear that you consider our study. It is encouraging that you also share the opinion, that this article is interesting, well-structured, well written and particularly scientific. The authors would like to thank you for your positive feedback on the manuscript. In the following we respond to your comments. We have accepted all comments and implemented.**

**Minor Revision**

However there are few points that still need further elaboration:

1. It would be helpful for the reader if the authors added a few lines explaining the terms used for the seven subscales (threat, centrality, controllable-by-self, controllable-by-others, uncontrollable, challenge and stressfulness) and what they denote in light of the study.

- **We explained the terms used for the seven subscales in the method section under the subchapter “The questionnaire based cross-sectional survey”.**

2. There is a very detailed representation of data in Table 1 regarding the demographic aspects of the study but no mention of these results in the discussion. It would be interesting to see how age, gender and the working arrangement of the dentists relate to the psychological and physical stress experienced by them when treating children with ASD. There is a clear majority of females in the study and its relation to the results must be highlighted in the discussion.

- **We took age, work experience and gender into account as possible factors influencing the stress experience.**

3. More references of studies done in other parts of the world can strengthen the discussion especially those studies where dentists having less experience are questioned for the study.

- **To account for the specific expertise in dealing with autistic patients, we have added another study.**

4. Line 65, the word should be ‘based’

- **We corrected the typo.**

5. Line 113, what is the meaning of ‘resp’ or is it a typo?

- **We corrected resp. into respectively.**

6. Line 338, a in place ‘of’

- **We corrected the typo.**

7. Line 393, ‘states’ instead of ‘statements’.

- **We corrected the typo.**
