# Supplementary material for: Is examining children and adolescents with autism spectrum disorders a challenge?—Measurement of Stress Appraisal (SAM) in German dentists with key expertise in paediatric dentistry
Source: PLoS One. 2022 Aug 3;17(8):e0271406. doi: 10.1371/journal.pone.0271406 (PMC9348685; doi:10.1371/journal.pone.0271406)
Supplement: Attachment — Submitted filename: Response to Reviewers.docx [file pone.0271406.s002.docx]

Dear Professor Kielbassa, dear reviewers,

we would like to thank you for the helpful notes and comments on our submitted manuscript. We hope that we were able to meet the editorial requirements of the journal as well as the needs of the reviewers to improve the manuscript in our revision.

**Comments from external reviewer:**

While I acknowledge the authors have made significant attempts to address my points and there are improvements to the manuscript, I am still of the opinion that the study lacks meaningful results due to coverage issues which have limited impact.

*Thank you very much for your review. Thank you for acknowledging our efforts to improve the manuscript based on your comments. With regard to the sample size and the generalization of the results to the population of German dentists with key interest and key expertise in paediatric dentistry or to general dentists, the impact of the results can certainly be regarded as limited. However since the question of stress vs. challenge has not yet been scientifically investigated, but has always been spoken of as a challenge based on experience, we assume that our manuscript has a scientific impact.*

*In lines 441 to 451 we address the fact that further research work of our scientific group will advance the validation process of the SAM in dentistry. Furthermore, we are working on the topic of examination of patients with ASD in dentistry in further surveys (441-451, page 22).*

*The results of the feasibility of the SAM are meaningful in our opinion, as they confirm similar results from other studies on the feasibility with a different sample. Cognition and processing of stress can be measured feasibly and with sufficient validity using the "Stress Appraisal Measure" (SAM) also in dentists*.

*Finally, we would like to point out that in the few studies on the subject of "attitudes of dentists to the treatment of children with ASD" also small sample sizes were reported. For example the study by Weil et al 2010 „Treating patients with autism spectrum disorder -SCDA members' attitudes and behavior“ only reported a sample size of 75 members of the society .*

Although the response rate to the survey is mentioned more explicitly as a key limitation in the study, and it is now mentioned appropriately in the abstract, reference to it is still missing from the results section. An overall response rate should be presented in the first line of the results section to ensure readers understand the context to the results and how many respondents they relate to, this is a key expectation in cross sectional studies. I am aware that many journals will not publish a cross sectional survey with such a low response rate and I am not convinced the authors responses adequately address the issue of quality in their approach.

*We presented the overall response rate (for the fully completed questionnaires and for all questionnaires started) in the first line of the results section (219-221, page 10).*

The results of the study are not particularly remarkable, as one would expect dentists with an interest in paediatric dentistry to experience less stress when treating children and adolescents with ASD. Again, this for me is a design flaw and the study might have applied an alternative design to elucidate the views of different type of dentists, which might of provided some greater insight into the issue. Indeed, the conclusion of the paper appears to recommend “special training sessions in special needs dentistry for dentists involved in the treatment of children and adolescents with ASD” but I am not sure the results of this study substantiate this point and it is not clear if the paper is recommending more training for paediatric dentists or the general dental population. I suspect it is the latter but that cannot be made based on the results of this study. This study provides only data on the utility of SAM in measuring stress response and the perceived levels of stress in a small sample of pediatric dentists.

*For incremental validation, we started with a small group of dentists (those with expertise in paediatric dentistry). If validation had not been successful in this group, further validation in larger and other groups of dentists from Germany would probably not have been necessary.*

*Now, our scientific working group is planning further surveys on this subject and same questions with general dentists and has already carried out surveys with dentists in the public health sector and also with people working in child and adolescent psychiatry, psychosomatic medicine and psychotherapy in Germany.*

*Our assumption is that general dentists experience a higher level of stress when treating children and adolescents with ASD than dentists with key expertise in paediatric dentistry. This must now be checked in one of the next steps.* *Nevertheless, we recommend, in the knowledge from the professional (clinic and research everyday life), that further training on this topic are recommended for both groups (general dentists and dentist with key expertise in paediatric dentistry) or for all dentists.*

6. Review Comments to the Author

Reviewer #1: With the help of the reviewers, this revised and re-submitted draft has been considerably improved, and is considered ready for external review.

*Thank you very much for your review.*

Reviewer #2: (No Response)

Reviewer #3: While the authors have made some improvements in the article however I still feel that there should be mention of similar studies done in other parts of the world as it gives a bigger picture of the challenges faced in dealing with children with Autism. If the the authors can highlight this point then it is acceptable for publication.

*Thank you very much for your review. We researched further studies on the topic and included them in the manuscript.*
